# Nail changes in connective tissue diseases: a study of 39 cases

**DOI:** 10.11604/pamj.2014.18.150.4637

**Published:** 2014-06-17

**Authors:** Imane Elmansour, Soumia Chiheb, Hakima Benchikhi

**Affiliations:** 1 Department of dermatology, Ibn Rushd University Hospital, Casablanca, Morocco

**Keywords:** Nail changes, connective tissue diseases, nail fold, Periungueal telangiectasia, longitudinal ridging, fingertips scars, peri ungueal erythema, ragged cuticle

## Abstract

The objective is to identify nail unit changes associated with connective tissue diseases (CTD) and evaluate their frequency. We carried a prospective study between March 2012 and March2013 in our department. All patients with CTD were included. A clinical examination of the fingernails was done by the same dermatologist. Nail features were noted and classified and photos taken. Thirty nine patients were enrolled including: 16 systemic sclerosis, 14 lupus erythematosus (SLE), 8 dermatomyositis (DM), 1 primary Sjorgen's syndrome. The mean age was 40 years old. The mean duration of the disease was 6 years. Nail unit changes were present in 27 patients (69%). The abnormalities observed were Longitidunal ridging in 11 patients, Peri ungueal erythema in 10 patients, Peri-ungual telangiectasia in 11 patients, Ragged cuticle in 10 patients fingertips scars in 9 patients, Increase of longitudinal curvature and beaking of the nail in 4 patients, Increase in transverse curvature in 4 patients, dyschromia of the proximal nail fold in 3 patients, Subungual hyperkeratosis in 3 patients, onycholysis in 2 patients, splinter haemorrhages in 3 patients, nail plate pigmentation in 2 patients, pseudoclubbing in 1 patient, macrolunula in 1 patients, Red lunulae in one patient, bluish- black discoloration of the nail plate in one patient. The proximal nailfold was found to be most sites affected.

## Introduction

Nail changes in connective tissue diseases (CTD) are common. Most of them are not specific however they may give an important clue to the diagnosis. Thus their knowledge is important for dermatologists and internists.

## Methods

Prospective study was carried in our department between Februaury 2012 and March 2013. All patients with connective tissue diseases (lupus, scleroderma,dermatomyositis, primary Sjorgen syndrome) were included. A detailed clinical examination (by the the naked eye) of the fingernails unit was done by the same dermatologist. Nail features were noted and classified. Photos were taken.

## Results

**Figure 1:**
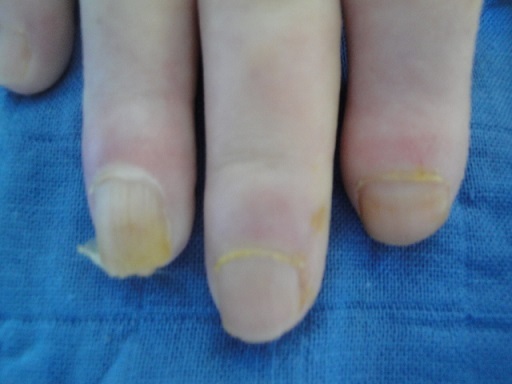
Longitudinal ridging, ragged cuticle and periungual telangiectasia in a patient with systemic sclerosis

**Figure 2:**
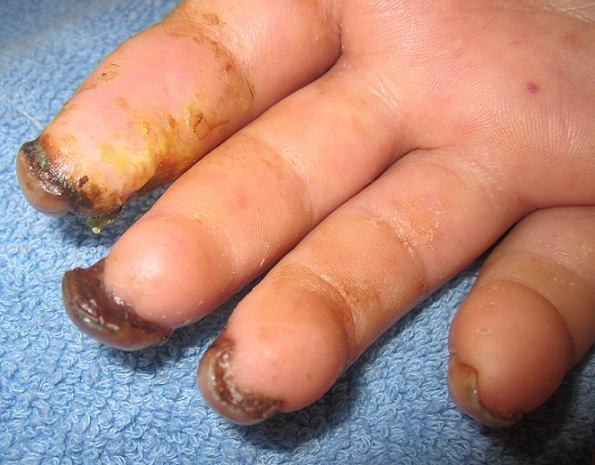
Beaking of the nail in a paient with systemic sclerosis

**Figure 3:**
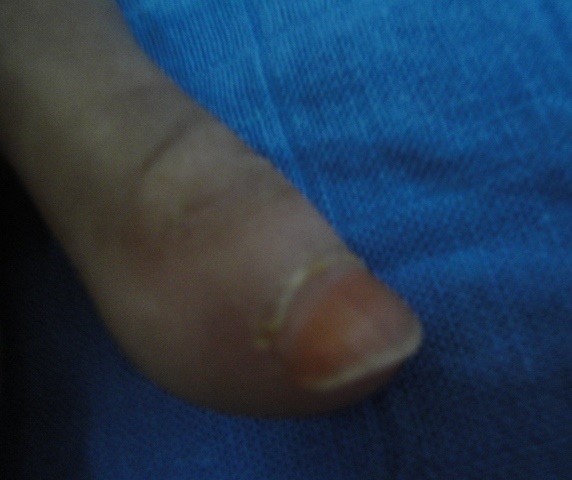
Increase of transversal curvature and ragged cuticle in a patient with systemic sclerosis

**Figure 4:**
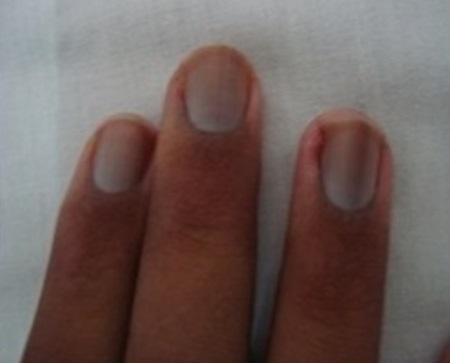
Bluish coloration of the nail plate in a patient with systemic lupus erythematosus

**Figure 5:**
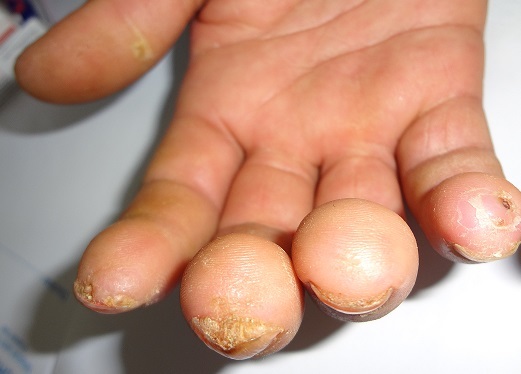
Subungueal keratosis in a patient with systemic lupus erythematosus

**Figure 6:**
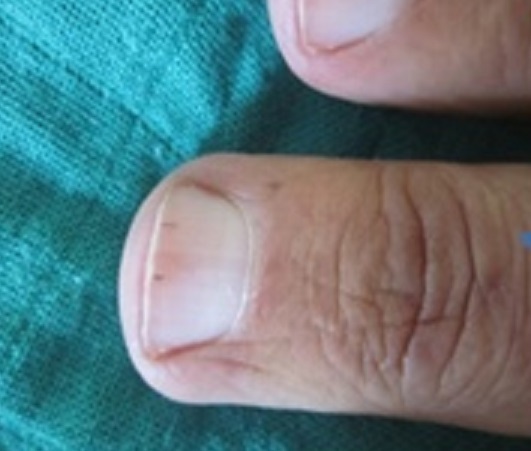
Splinter haemorrhage in a patient with a clinically active systemic lupus erythematosus

**Figure 7:**
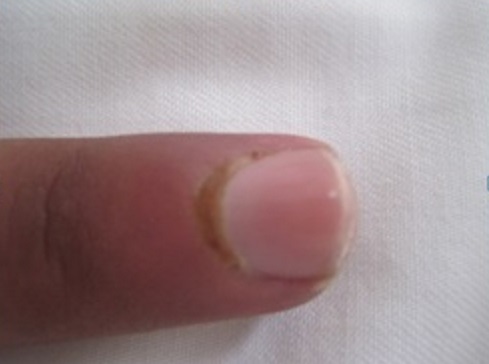
Ragged cuticle and periungueal erythema in a patient with dermatomyositis

Thirty nine patients were enrolled in the study (33 females, 6 males) including: 16 systemic sclerosis, 14 lupus erythematosus (SLE), 8 dermatomyositis, one primary Sjorgen′s syndrome. The mean age was 40 years old. The mean duration of the disease was 6 years. Nail unit changes were present in 27 patients (69%). Out of 16 patients with scleroderma, 14 had nail changes. The abnormalities observed were: nail fold telangiectasia (9patients), ragged cuticles (6 patients), Longitidunal ridging (4 patients) (Figure 1:), Increase of longitudinal curvature, and beaking of the nail (4patients) (Figure 2:), increase in transverse curvature(4 patients) (Figure 3:), longitudinal melanonychia (2patients) Ventral pterygium (2patients), pseudoclubbing(1patient), macrolunula (1patient), subungual keratosis in one patient. Ten patients had fingertip scars and two had digital necrosis. Seven patients with SLE showed nail changes, proximal nail-fold erythema was noted in 4 patients, Longitudinal ridging in 4 patients, bluish- black discoloration of the nail plate in one patient (Figure 4:), onycholysis in 2patients, subungual hyperkeratosis in 2patients (Figure 5:) splinter haemorrhages in 3 patients (Figure 6:) and red lunula in one patient. All the patients with nail changes in dermatomyositis group (6 patients) had nail fold erythema. Four of them had ragged, hyperkeratosic cuticles (Figure 7:), 2 had nail fold telagiectasia and 3had longitudinal ridging.

## Discussion

Nail unit changes were present in 27 patients (69, 2%). The proximal nailfold was found as the most site affected. The aspects most frequently seen were nail fold telangiectasia and longitudinal ridging. These results were similar to previous studies [1, 2] (Table 1). In scleroderma patients, Nail Fold Telangiectasia was the abnormality most frequently seen. The other changes observed were longitudinal ridging, ragged cuticle, Increase of longitudinal curvature, increase in transverse curvature and beaking of the nail, marcrolunula, pseudoclubbing. All patients had Raynaud's phenomen and digital scars. This finding was consistent with other studies Axel F G von Bierbrauer [3] performed nail fold biopsies of 24 patients with scleroderma. The main finding were perivascular round cell (70,8%), mast cells and fibroblasts (45,8%) infiltrations, increased amounts of connective tissue(75%), signs of microangiopathy (87-5%), splitting of the L-basallamina (66,7%), perivascular oedema (41,7%), deposits of complement C3(66%),IgG(62%), or both in the vessel wall and in the perivascular region, perivascular deposits of amorphic material (25%). These features are not specific. Similar histopathological changes have been reported in lupus erythematosus,, dermatomyositis, mixed connective tissue disease, and diabetes [3–5].

**Table 1:** Nail changes in connective tissue diseases in different studies

	Systemic sclerosis			Systemic lupus erythematosus			Dermatomyositis/polymyositis		
Nail changes (%)	This study n=16	PA Nabil n=7	Tunc etal n=39	This study n=14	PA Nabil n=18	Tunc et al n=56	This study n=8	PA Nabil n=1	Tunc et al n=13
Nail fold telangiectasia	56	57	-	-	11.11	-	25	1(100)	4
Longitidunal ridging	25	28.6	37	28.5	22	52	37.5	13	13
Fingertips scars	56.2	42.86	-	-	-	-	-	-	-
Peri ungueal erythema	-	-	2	28.57	54.54	12	50	-	7.7
Ragged cuticle/ cuticar hypertrophy	37.5	42.86	-	-	16.6	-	50	-	-
Increase of longitudinal curvature	25	-	12	-	-	1	-		-
Increase in transverse curvature and beaking of the nail	25		10	-	-	-	-	-	-
Subungual hyperkeratosis	-	-	-	14.2	-	-	-	-	-
Onycholysis	-	-	-	14.2	-	-	-	-	-
Nail plate pigmentation	12.5	-	-	7.1		-	-	-	-
Ventral pterygium	12.5	-	-				-	-	-
Splinter haemorrhages	-		7	21.4	-	11	-	-	3
Pseudoclubbing	6.25	-	-	-	-	-	-	-	-
Dyschromia of the proximal nail fold	18.75	28.57	-	-	22		-	-	-
Macrolunula	6.25	-	-	-	-	-	-	-	-
Red lunulae	-	-	10.3	7.1	-	7.1	-	-	15.4

In a case control study [2], comparing nail changes in patients with CTD to 2 healthy groups, Capillary loops and splinter hemorrhages were frequent significantly in scleroderma patients, whereas increase in longitudinal and transverse curvature were significantly frequent only in scleroderma patients. Increase in transverse curvature of patients with systemic sclerosis (SSc) was found to be associated significantly with disease activity. Other studies showed that there is an association between the degree of nail fold capillary abnormality (as detected by capillaroscopy) and internal organ involvement and mortality in scleroderma patients [6, 7]. Patients with Raynaud′s disease are likely to develop systemic manifestations later in the course of the disease when they have associated nail fold telangiactasia [8]. Commonest nail change observed in our patients with SLE was proximal nail-fold erythema. This finding was consistent with other studies. Other changes observed were Longitidunal ridging, splinter haemorrhages, bluish black discoloration of the nail plate, onycholysis, subungual hyperkeratosis and red lunula. Ventral pterygium, Thin nail-plate, beau line, longitudinal melanonychia, pincer nail deformity Punctate or striate leuconychia, nail pitting)Lupus erythematosus unguium mutilanswere also reported to be found in patients with SLE.[2, 9–11] In the study of SE Tunc, Splinter haemorrhage in fingernails of patients with SLE was found to be associated significantly with disease activity [2]. Urowitz MB found that the incidence of mucous membrane ulcerations and Raynaud′s phenomenon was in SLE patients who had nail abnomalities [12].

The different types of nail lesions encountered in patients with LE are classified into those that are histologically specific for LE (Discoid LE of the nail unit Hypertrophic LE Chilblains LE Lupus erythematosus unguium mutilans) and those that are not (Leuconychia, Nail pitting, ridging, onycholysis, nail dyschromia, nail fold erythema, red lunulae, nail fold hyperkeratosis, ragged cuticles, and splinter haemorrhages, vasculopathic lesions of the nail unit, pterygium inversum unguis.[9] Direct immunofluorescence of proximal nail fold changes in SLE shows the typical immune deposit pattern ofLE (lupus band) at the dermal-epidermal. In dermatomyositis patients, abnormalities seen were periungual erythema, hyperkeratosic, ragged cuticles, nail fold telangiectasia and longitudinal ridging. Other works have reported splinter haemorrhages, capillary loops in proximal nailfold, red lunulae, loss of several toenails [13–16] Cuticular changes (Thickening and hyperkeratosis hardness, roughness) are common in DM. These alterations, described by Keining in 1939 [13], are not pathognomonic; similar cuticular changes have been reported in scleroderma and lupus erythematous. Although a characteristic feature of DM is the association of cuticular changes with periungual erythema,telangiectasia and hemorrhages [14]. All our patients with cuticular changes had periungueal erythema. Samitz reported that resolution of cuticular changes occurred with improvement of the patients dermatomyositis and reappeared with recurrences of the disease paralleling its severity [15].

Tr Ekmekci, noticed that cuticles were the first site healed in a patient with dermatomyositis and considered that it may be an indicator of clinical remission [17]. The majority of nail complex changes associated with connective tissue diseases represent non specific reaction pattern. Their mechanism is unknown, but it is probably due to vasculitic process [1, 2].

## Conclusion

Knowledge of nail changes in CTD may provide clues to the diagnosis, differenciate them from other onychopathies such as onychomycosis and provides prognostic information [1]. Especially when associated with other diagnostic tools like biopsy of the nailfold and capillaroscopy or dermoscopy.
